# Bis{1-[(*E*)-(2-methyl­phen­yl)diazen­yl]-2-naphtho­lato}palladium(II)

**DOI:** 10.1107/S1600536810028916

**Published:** 2010-07-24

**Authors:** Meng-Ling Lin, Chen-Yen Tsai, Chen-Yu Li, Bor-Hunn Huang, Bao-Tsan Ko

**Affiliations:** a Department of Chemistry, Chung Yuan Christian University, Chung-Li 320, Taiwan; b R&D department, Min Chung Technology, Hsinchu City 300, Taiwan

## Abstract

In the title compound, [Pd(C_17_H_13_N_2_O)_2_], the Pd^II^ atom is tetra­coordinated by two N atoms and two O atoms from two bidentate methylphenyl­diazenylnaphtolate ligands, forming a square-planar complex. The two N atoms and two O atoms around the Pd^II^ atom are *trans* to each other (as the Pd^II^ atom lies on a crystallographic inversion centre) with O—Pd—N bond angles of 89.60 (11) and 90.40 (11)°. The distances between the Pd^II^ atom and the coordinated O and N atoms are 1.966 (3) and 2.009 (3) Å, respectively.

## Related literature

For the Suzuki cross-coupling reactions of palladium complexes with *N,O*-bidentate ligands or imine–phenol ligands, see: Lai *et al.* (2005 ▶). For the synthesis and characterization of a bis­(phen­oxy­ketimine) Pd(II) complex, see: Brayton *et al.* (2009 ▶). For a related structure: see: Tsai *et al.* (2009 ▶).
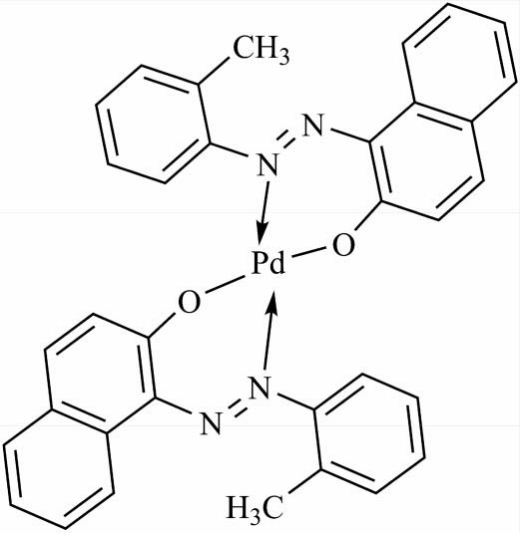


## Experimental

### 

#### Crystal data

[Pd(C_17_H_13_N_2_O)_2_]
*M* *_r_* = 628.99
Monoclinic, 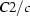

*a* = 22.9997 (7) Å
*b* = 4.8374 (2) Å
*c* = 24.7294 (7) Å
β = 94.151 (2)°
*V* = 2744.15 (16) Å^3^
*Z* = 4
Mo *K*α radiation
μ = 0.72 mm^−1^
*T* = 296 K
0.53 × 0.28 × 0.15 mm

#### Data collection

Bruker APEXII CCD diffractometer
Absorption correction: multi-scan (*SADABS*; Bruker, 2008 ▶) *T*_min_ = 0.783, *T*_max_ = 0.900
12830 measured reflections
3412 independent reflections
2463 reflections with *I* > 2σ(*I*)
*R*_int_ = 0.029

#### Refinement

*R*[*F* ^2^ > 2σ(*F* ^2^)] = 0.034
*wR*(*F* ^2^) = 0.091
*S* = 1.01
3412 reflections
187 parameters
H-atom parameters constrained
Δρ_max_ = 0.57 e Å^−3^
Δρ_min_ = −0.39 e Å^−3^

### 

Data collection: *APEX2* (Bruker, 2008 ▶); cell refinement: *SAINT-Plus* (Bruker, 2008 ▶); data reduction: *SAINT-Plus*; program(s) used to solve structure: *SHELXTL* (Sheldrick, 2008 ▶); program(s) used to refine structure: *SHELXTL*; molecular graphics: *DIAMOND* (Brandenburg, 2006 ▶); software used to prepare material for publication: *SHELXTL*.

## Supplementary Material

Crystal structure: contains datablocks I, global. DOI: 10.1107/S1600536810028916/nk2047sup1.cif

Structure factors: contains datablocks I. DOI: 10.1107/S1600536810028916/nk2047Isup2.hkl

Additional supplementary materials:  crystallographic information; 3D view; checkCIF report

## Figures and Tables

**Fig. 1.**
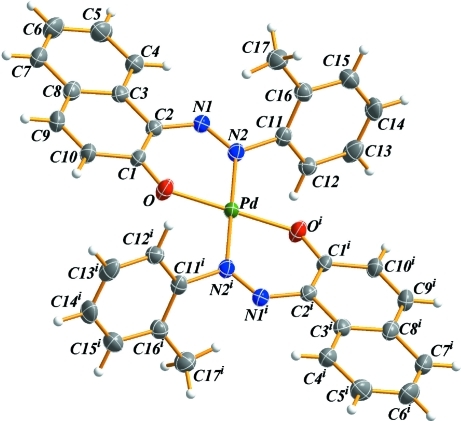
A view of the I with the atom numbering scheme. Displacement ellipsoids are drawn at the 30% probability level. Symmetry operator: i, -x+1/2, -y+3/2, -z+2.

## supplementary crystallographic information

### Comment

1-Phenylazo-2-naphtol (PAN-H) derivatives are widely used as an orange-red appearance for the additive of colour waxes, oil, petrol, solvents and polishes. In term of coordination chemistry, the phenylazo-naphtolate group can provide *N,O*-bidentate chelation to stabilize the transition metal or main group metal complexes. Recently, Lai *et al.* (2005) reported the palladium complexes supported by *N,O*-bidentate ligands and the imine-phenol ligands in the presence of Pd(OAc)_2_ have been demonstrated effectively to catalyze Suzuki cross-coupling reactions. Most recently, the air- and moisture-stable bis(phenoxyketimine) Pd(II) complex has been synthesized and characterized (Brayton *et al.*, 2009). In addition, its activity as the precatalyst in the Suzuki–Miyaura crosscoupling reaction has been studied, and it catalyzes the coupling of unactivated aryl bromides with boronic acids in good yields under mild temperature and short reaction time. Therefore, our group is interested in the synthesis and preparation of palladium complexes derived from *N, O*-bidentate ligands. For example, our group has successfully synthesized and structural characterized the Pd complex (II) with 4-methyl-2-(2*H*-benzotriazol-2-yl)-phenolate ligands (Tsai *et al.*, 2009). We report herein the synthesis and crystal structure of *N, O*-bidentate phenylazo-naphtolate ligands incorporated Pd^II^ complex (I), a potential catalyst for palladium-catalyzed Suzuki cross-coupling reactions (Scheme 1).

The solid structure of (I) reveals a monomeric Pd^II^ complex (Fig. 1) containing two six-membered rings coordinated from these two *N, O*-bidentate phenylazo-naphtolate ligands. It was found that the asymmetric unit has one half of molecule in which the Pd atom lies on a centre of symmetry. The geometry around Pd atom is tetra-coordinated with a normal square planar environment in which two nitrogen atoms and two oxygen atoms are coplanar. The two N atoms and two O atoms around Pd atom are *trans* to each other with an O—Pd—N2 bond angle of 89.60 (11)° and O—Pd—N2^i^ of 90.40 (11)°. The distances between the Pd atom and O and N2 are 1.966 (3) Å, 2.009 (3) Å, respectively. These bond distances and angles are similar to those found in the crystal structure of bis[4-methyl-2-(2*H*-benzotriazol-2-yl)phenolato]Palladium (II) (Tsai *et al.*, 2009).

### Experimental

The title compound (I) was synthesized by the following procedures:

(*E*)-1-(*o*-tolyldiazenyl)naphthalen-2-ol (0.52 g, 2.0 mmol) and Pd(OAc)_2_ (0.22 g, 1.0 mmol) was stirred at 298 K in THF (25 ml) for 24 h. Volatile materials were removed under vacuum and the residue was washed twice from hexane solution to give dark purple solids. The resulting solids were crystallized from CH_2_Cl_2_/Hexane (1:5) solution to yield purple crystals.

### Refinement

The H atoms were placed in idealized positions and constrained to ride on their parent atoms, with C–H = 0.93 Å with *U_iso_*(H) = 1.2 *U*_eq_(C) for phenyl hydrogen; 0.96 Å with *U*_iso_(H) = 1.5 *U*_eq_(C) for CH_3_ group.

### Crystal data

**Table:**

[Pd(C_17_H_13_N_2_O)_2_]	*F*(000) = 1280
*M**_r_* = 628.99	*D*_x_ = 1.522 Mg m^−^^3^
Monoclinic, *C*2/*c*	Mo *K*α radiation, λ = 0.71073 Å
Hall symbol: -C 2yc	Cell parameters from 7356 reflections
*a* = 22.9997 (7) Å	θ = 2.3–28.2°
*b* = 4.8374 (2) Å	µ = 0.72 mm^−^^1^
*c* = 24.7294 (7) Å	*T* = 296 K
β = 94.151 (2)°	Block, purple
*V* = 2744.15 (16) Å^3^	0.53 × 0.28 × 0.15 mm
*Z* = 4	

### Data collection

**Table:**

Bruker APEXII CCD diffractometer	3412 independent reflections
Radiation source: fine-focus sealed tube	2463 reflections with *I* > 2σ(*I*)
graphite	*R*_int_ = 0.029
Detector resolution: 8.3333 pixels mm^-1^	θ_max_ = 28.3°, θ_min_ = 1.7°
φ and ω scans	*h* = −29→30
Absorption correction: multi-scan (*SADABS*; Bruker, 2008)	*k* = −6→6
*T*_min_ = 0.783, *T*_max_ = 0.900	*l* = −32→32
12830 measured reflections	

### Refinement

**Table:**

Refinement on *F*^2^	Primary atom site location: structure-invariant direct methods
Least-squares matrix: full	Secondary atom site location: difference Fourier map
*R*[*F*^2^ > 2σ(*F*^2^)] = 0.034	Hydrogen site location: inferred from neighbouring sites
*wR*(*F*^2^) = 0.091	H-atom parameters constrained
*S* = 1.00	*w* = 1/[σ^2^(*F*_o_^2^) + (0.0308*P*)^2^ + 8.4214*P*] where *P* = (*F*_o_^2^ + 2*F*_c_^2^)/3
3412 reflections	(Δ/σ)_max_ < 0.001
187 parameters	Δρ_max_ = 0.57 e Å^−^^3^
0 restraints	Δρ_min_ = −0.39 e Å^−^^3^

### Special details

**Table:**

Geometry. All e.s.d.'s (except the e.s.d. in the dihedral angle between two l.s. planes) are estimated using the full covariance matrix. The cell e.s.d.'s are taken into account individually in the estimation of e.s.d.'s in distances, angles and torsion angles; correlations between e.s.d.'s in cell parameters are only used when they are defined by crystal symmetry. An approximate (isotropic) treatment of cell e.s.d.'s is used for estimating e.s.d.'s involving l.s. planes.
Refinement. Refinement of *F*^2^ against ALL reflections. The weighted *R*-factor *wR* and goodness of fit *S* are based on *F*^2^, conventional *R*-factors *R* are based on *F*, with *F* set to zero for negative *F*^2^. The threshold expression of *F*^2^ > σ(*F*^2^) is used only for calculating *R*-factors(gt) *etc*. and is not relevant to the choice of reflections for refinement. *R*-factors based on *F*^2^ are statistically about twice as large as those based on *F*, and *R*– factors based on ALL data will be even larger.

### Fractional atomic coordinates and isotropic or equivalent isotropic displacement parameters (Å^2^)

**Table:**

	*x*	*y*	*z*	*U*_iso_*/*U*_eq_	
Pd	0.2500	0.7500	1.0000	0.03581 (10)	
O	0.25231 (10)	0.8334 (5)	0.92226 (8)	0.0515 (6)	
N1	0.34684 (10)	0.4378 (5)	0.95474 (9)	0.0395 (5)	
N2	0.32263 (10)	0.5212 (5)	0.99676 (9)	0.0383 (5)	
C1	0.28284 (12)	0.7041 (7)	0.88889 (11)	0.0412 (7)	
C2	0.32620 (11)	0.5049 (7)	0.90302 (10)	0.0383 (6)	
C3	0.35764 (12)	0.3717 (7)	0.86127 (11)	0.0418 (7)	
C4	0.40016 (14)	0.1696 (8)	0.87324 (13)	0.0528 (8)	
H4A	0.4087	0.1152	0.9090	0.063*	
C5	0.42950 (15)	0.0503 (9)	0.83250 (15)	0.0667 (11)	
H5A	0.4575	−0.0845	0.8410	0.080*	
C6	0.41752 (17)	0.1303 (11)	0.77874 (15)	0.0745 (12)	
H6A	0.4381	0.0519	0.7516	0.089*	
C7	0.37581 (16)	0.3227 (9)	0.76598 (13)	0.0625 (10)	
H7A	0.3675	0.3721	0.7299	0.075*	
C8	0.34474 (13)	0.4492 (8)	0.80650 (12)	0.0485 (8)	
C9	0.30133 (14)	0.6511 (8)	0.79407 (12)	0.0539 (8)	
H9A	0.2930	0.7018	0.7581	0.065*	
C10	0.27170 (14)	0.7722 (7)	0.83272 (12)	0.0492 (8)	
H10A	0.2433	0.9031	0.8227	0.059*	
C11	0.35700 (12)	0.4466 (6)	1.04613 (11)	0.0388 (6)	
C12	0.33396 (14)	0.2599 (7)	1.08096 (12)	0.0457 (7)	
H12A	0.2972	0.1845	1.0727	0.055*	
C13	0.36591 (17)	0.1857 (8)	1.12820 (14)	0.0611 (10)	
H13A	0.3510	0.0577	1.1516	0.073*	
C14	0.41908 (16)	0.3001 (9)	1.14040 (14)	0.0651 (11)	
H14A	0.4403	0.2519	1.1725	0.078*	
C15	0.44195 (14)	0.4868 (9)	1.10574 (13)	0.0596 (9)	
H15A	0.4786	0.5620	1.1148	0.071*	
C16	0.41154 (12)	0.5661 (7)	1.05728 (12)	0.0440 (7)	
C17	0.43715 (16)	0.7699 (8)	1.02047 (16)	0.0627 (10)	
H17A	0.4105	0.8002	0.9893	0.094*	
H17B	0.4733	0.6992	1.0090	0.094*	
H17C	0.4441	0.9413	1.0394	0.094*	

### Atomic displacement parameters (Å^2^)

**Table:**

	*U*^11^	*U*^22^	*U*^33^	*U*^12^	*U*^13^	*U*^23^
Pd	0.03590 (16)	0.04187 (19)	0.02960 (14)	0.00156 (14)	0.00184 (10)	0.00060 (13)
O	0.0571 (13)	0.0640 (16)	0.0342 (10)	0.0177 (11)	0.0081 (9)	0.0076 (10)
N1	0.0398 (12)	0.0433 (15)	0.0354 (11)	0.0000 (11)	0.0026 (9)	−0.0019 (11)
N2	0.0404 (11)	0.0421 (15)	0.0323 (11)	0.0021 (11)	0.0021 (9)	0.0013 (10)
C1	0.0402 (14)	0.052 (2)	0.0315 (13)	−0.0049 (13)	0.0044 (10)	0.0024 (12)
C2	0.0361 (13)	0.0445 (18)	0.0347 (13)	−0.0046 (13)	0.0039 (10)	−0.0028 (12)
C3	0.0395 (14)	0.0472 (18)	0.0391 (14)	−0.0061 (14)	0.0050 (11)	−0.0055 (14)
C4	0.0454 (16)	0.066 (2)	0.0477 (17)	0.0004 (16)	0.0053 (13)	−0.0095 (16)
C5	0.0541 (19)	0.079 (3)	0.068 (2)	0.010 (2)	0.0079 (16)	−0.021 (2)
C6	0.064 (2)	0.104 (3)	0.057 (2)	0.000 (2)	0.0198 (18)	−0.029 (2)
C7	0.065 (2)	0.085 (3)	0.0382 (16)	−0.011 (2)	0.0101 (15)	−0.0117 (17)
C8	0.0511 (16)	0.057 (2)	0.0376 (14)	−0.0127 (16)	0.0069 (12)	−0.0082 (14)
C9	0.0611 (19)	0.069 (2)	0.0310 (14)	−0.0072 (18)	0.0005 (13)	0.0014 (15)
C10	0.0522 (17)	0.060 (2)	0.0348 (14)	0.0043 (16)	0.0003 (12)	0.0058 (15)
C11	0.0445 (14)	0.0389 (17)	0.0331 (13)	0.0084 (13)	0.0033 (11)	0.0005 (12)
C12	0.0480 (16)	0.0443 (18)	0.0450 (15)	0.0010 (15)	0.0046 (12)	0.0043 (15)
C13	0.071 (2)	0.065 (3)	0.0487 (18)	0.0165 (19)	0.0145 (16)	0.0212 (17)
C14	0.062 (2)	0.092 (3)	0.0404 (16)	0.020 (2)	−0.0032 (15)	0.0148 (18)
C15	0.0460 (17)	0.078 (3)	0.0535 (19)	0.0068 (18)	−0.0068 (14)	−0.0013 (19)
C16	0.0412 (14)	0.048 (2)	0.0431 (15)	0.0036 (14)	0.0017 (12)	0.0015 (14)
C17	0.0535 (19)	0.068 (3)	0.067 (2)	−0.0060 (18)	0.0039 (16)	0.0064 (19)

### Geometric parameters (Å, °)

**Table:**

Pd—O	1.9687 (19)	C7—H7A	0.9300
Pd—O^i^	1.9688 (19)	C8—C9	1.414 (5)
Pd—N2	2.010 (2)	C9—C10	1.347 (5)
Pd—N2^i^	2.010 (2)	C9—H9A	0.9300
O—C1	1.284 (4)	C10—H10A	0.9300
N1—N2	1.279 (3)	C11—C12	1.380 (4)
N1—C2	1.371 (3)	C11—C16	1.390 (4)
N2—C11	1.451 (3)	C12—C13	1.382 (4)
C1—C2	1.412 (4)	C12—H12A	0.9300
C1—C10	1.433 (4)	C13—C14	1.356 (5)
C2—C3	1.453 (4)	C13—H13A	0.9300
C3—C4	1.399 (5)	C14—C15	1.375 (5)
C3—C8	1.416 (4)	C14—H14A	0.9300
C4—C5	1.379 (5)	C15—C16	1.397 (4)
C4—H4A	0.9300	C15—H15A	0.9300
C5—C6	1.393 (5)	C16—C17	1.492 (5)
C5—H5A	0.9300	C17—H17A	0.9600
C6—C7	1.357 (6)	C17—H17B	0.9600
C6—H6A	0.9300	C17—H17C	0.9600
C7—C8	1.412 (4)		
			
O—Pd—O^i^	180.00 (13)	C7—C8—C3	118.9 (3)
O—Pd—N2	89.58 (9)	C9—C8—C3	119.1 (3)
O^i^—Pd—N2	90.43 (9)	C10—C9—C8	122.1 (3)
O—Pd—N2^i^	90.43 (9)	C10—C9—H9A	119.0
O^i^—Pd—N2^i^	89.57 (9)	C8—C9—H9A	119.0
N2—Pd—N2^i^	180.00 (14)	C9—C10—C1	121.6 (3)
C1—O—Pd	125.6 (2)	C9—C10—H10A	119.2
N2—N1—C2	122.8 (2)	C1—C10—H10A	119.2
N1—N2—C11	111.3 (2)	C12—C11—C16	122.0 (3)
N1—N2—Pd	128.15 (18)	C12—C11—N2	118.5 (3)
C11—N2—Pd	120.46 (17)	C16—C11—N2	119.5 (3)
O—C1—C2	125.6 (3)	C11—C12—C13	119.5 (3)
O—C1—C10	116.3 (3)	C11—C12—H12A	120.2
C2—C1—C10	118.0 (3)	C13—C12—H12A	120.2
N1—C2—C1	125.7 (3)	C14—C13—C12	119.9 (3)
N1—C2—C3	113.7 (3)	C14—C13—H13A	120.1
C1—C2—C3	120.4 (3)	C12—C13—H13A	120.1
C4—C3—C8	118.8 (3)	C13—C14—C15	120.6 (3)
C4—C3—C2	122.4 (3)	C13—C14—H14A	119.7
C8—C3—C2	118.8 (3)	C15—C14—H14A	119.7
C5—C4—C3	120.6 (3)	C14—C15—C16	121.6 (3)
C5—C4—H4A	119.7	C14—C15—H15A	119.2
C3—C4—H4A	119.7	C16—C15—H15A	119.2
C4—C5—C6	120.5 (4)	C11—C16—C15	116.4 (3)
C4—C5—H5A	119.7	C11—C16—C17	123.0 (3)
C6—C5—H5A	119.7	C15—C16—C17	120.6 (3)
C7—C6—C5	120.0 (3)	C16—C17—H17A	109.5
C7—C6—H6A	120.0	C16—C17—H17B	109.5
C5—C6—H6A	120.0	H17A—C17—H17B	109.5
C6—C7—C8	121.2 (3)	C16—C17—H17C	109.5
C6—C7—H7A	119.4	H17A—C17—H17C	109.5
C8—C7—H7A	119.4	H17B—C17—H17C	109.5
C7—C8—C9	122.0 (3)		
			
N2—Pd—O—C1	15.3 (3)	C6—C7—C8—C3	−0.1 (6)
N2^i^—Pd—O—C1	−164.7 (3)	C4—C3—C8—C7	−0.9 (5)
C2—N1—N2—C11	−173.5 (3)	C2—C3—C8—C7	179.2 (3)
C2—N1—N2—Pd	2.2 (4)	C4—C3—C8—C9	179.3 (3)
O—Pd—N2—N1	−12.3 (3)	C2—C3—C8—C9	−0.7 (5)
O^i^—Pd—N2—N1	167.7 (3)	C7—C8—C9—C10	−179.9 (3)
O—Pd—N2—C11	163.1 (2)	C3—C8—C9—C10	−0.1 (5)
O^i^—Pd—N2—C11	−16.9 (2)	C8—C9—C10—C1	0.4 (5)
Pd—O—C1—C2	−9.3 (5)	O—C1—C10—C9	179.6 (3)
Pd—O—C1—C10	171.2 (2)	C2—C1—C10—C9	0.1 (5)
N2—N1—C2—C1	10.7 (5)	N1—N2—C11—C12	−114.3 (3)
N2—N1—C2—C3	−175.7 (3)	Pd—N2—C11—C12	69.6 (3)
O—C1—C2—N1	−7.1 (5)	N1—N2—C11—C16	66.3 (4)
C10—C1—C2—N1	172.4 (3)	Pd—N2—C11—C16	−109.8 (3)
O—C1—C2—C3	179.7 (3)	C16—C11—C12—C13	−0.7 (5)
C10—C1—C2—C3	−0.9 (4)	N2—C11—C12—C13	180.0 (3)
N1—C2—C3—C4	7.2 (4)	C11—C12—C13—C14	1.1 (5)
C1—C2—C3—C4	−178.8 (3)	C12—C13—C14—C15	−0.9 (6)
N1—C2—C3—C8	−172.9 (3)	C13—C14—C15—C16	0.3 (6)
C1—C2—C3—C8	1.2 (4)	C12—C11—C16—C15	0.1 (5)
C8—C3—C4—C5	0.8 (5)	N2—C11—C16—C15	179.4 (3)
C2—C3—C4—C5	−179.3 (3)	C12—C11—C16—C17	−179.5 (3)
C3—C4—C5—C6	0.4 (6)	N2—C11—C16—C17	−0.2 (5)
C4—C5—C6—C7	−1.4 (7)	C14—C15—C16—C11	0.1 (5)
C5—C6—C7—C8	1.3 (7)	C14—C15—C16—C17	179.7 (4)
C6—C7—C8—C9	179.7 (4)		

Symmetry codes: (i) −*x*+1/2, −*y*+3/2, −*z*+2.
